# 3D Printing for Cardiovascular Applications: From End-to-End Processes to Emerging Developments

**DOI:** 10.1007/s10439-021-02784-1

**Published:** 2021-05-17

**Authors:** Ramtin Gharleghi, Claire A. Dessalles, Ronil Lal, Sinead McCraith, Kiran Sarathy, Nigel Jepson, James Otton, Abdul I. Barakat, Susann Beier

**Affiliations:** 1 Faculty of Engineering, School of Mechanical and Manufacturing, UNSW, Sydney, Australia; 2 LadHyX - UMR 7646, École Polytechnique, Palaiseau, France; 3 Prince of Wales Hospital, Sydney, Australia; 4 Prince of Wales Clinical School of Medicine, UNSW, Sydney, Australia; 5 Department of Cardiology, Liverpool Hospital, Sydney, Australia

**Keywords:** Medical technology, Additive manufacturing, Rapid prototyping, Bioprinting, Tissue engineering, Virtual reality

## Abstract

3D printing as a means of fabrication has seen increasing applications in medicine in the last decade, becoming invaluable for cardiovascular applications. This rapidly developing technology has had a significant impact on cardiovascular research, its clinical translation and education. It has expanded our understanding of the cardiovascular system resulting in better devices, tools and consequently improved patient outcomes. This review discusses the latest developments and future directions of generating medical replicas (‘phantoms’) for use in the cardiovascular field, detailing the end-to-end process from medical imaging to capture structures of interest, to production and use of 3D printed models. We provide comparisons of available imaging modalities and overview of segmentation and post-processing techniques to process images for printing, detailed exploration of latest 3D printing methods and materials, and a comprehensive, up-to-date review of milestone applications and their impact within the cardiovascular domain across research, clinical use and education. We then provide an in-depth exploration of future technologies and innovations around these methods, capturing opportunities and emerging directions across increasingly realistic representations, bioprinting and tissue engineering, and complementary virtual and mixed reality solutions. The next generation of 3D printing techniques allow patient-specific models that are increasingly realistic, replicating properties, anatomy and function.

## Background

Clinical diagnosis has increasingly shifted from being invasive towards being informative. Advances in medical imaging have played a pivotal role in cardiovascular care to date, providing indispensable support in clinical settings. Yet, imaging modalities remain limited due to the lack of dynamic interaction, tactile feedback and layered structural information even when multiple views and color overlays are available. 3D printing enables physical manufacturing or printing of 3D replicas of the imaged tissue in a layer-by-layer fashion, replicating structure, behavior and even function.^102^,^137^,^145^ The underlying knowledge of the patient’s anatomy is imperative to the success of the often complex cardiovascular intervention, which is compounded by the fact that cardiovascular anatomy itself varies widely within a population.^84^,^97^ The ability to not just better visualize, but also to touch and closely examine with scaling, labeling and color options, and even disassemble a patient’s tissue in its anatomy and pathology offers unprecedented opportunities which is not captured by standard modalities and the workflow of flat-screen, commonly grayscale images.^134^ 3D printing and related technologies have facilitated the applications ranging from more traditional educational and assisted communication purposes, 7,152,160 to increasingly futuristic direct clinically-relevant training, procedural planning and device optimization. This is recognized as critical since for most clinical interventions a repair rather than a complete structural replacement is desired (stenting versus bypass surgery, mitral valve repair versus replacement *etc*.), and the potential for greater treatment success with direct benefits for patient well-being is concrete. Commercially available 3D printing services^1^ and comprehensive open-source libraries^104^,^151^ have paved the way for increasing integration of 3D printing in hospitals. 3D printing has also found use in research, being used for analyzing a range of models such as aorta, coronary arteries and whole heart, for both normal and diseased physiology.^122^ Together this gave rise to significant device innovation and new physiological and patho-physiological insights in recent years.^100^

3D printing, also called additive manufacturing (AM), traditionally refers to the layer-by-layer build-up of non-biological material such as laser-solidified polymers or alloys.^16^ The emerging field of 3D bioprinting uses similar methods with biological materials such as living cells with the vision to 1 day regenerate whole structures or organs as an integral part of tissue engineering.^68^ Whilst the use of depositing/printing biological materials remains a developing field with major remaining challenges, it has gained momentum in recent years with significant breakthroughs, especially for vascular structures. 3D printing industry has experienced a rapid world-wide revenue growth with a projected market value of $50 billion USD by 2025.^52^ The advances of technologies under the umbrella of 3D printing, as well as virtual and mixed reality technologies, will be a major factor in accelerating the trend towards personalized and precise medical care, especially in cardiology.^154^

In this review, we first introduce the general concepts and associated processes involved in 3D printing from cardiovascular imaging to phantom production. Specifically, we collated detailed information and developed significant reader guidance on all aspects of associated workflows, including image acquisition, segmentation, and registration options. We then explain and compare additive manufacturing technologies, before discussing milestone applications in the cardiovascular field across education, research and clinical practice. Then we explore promising future developments in 3D printing, and its related and competing technologies of tissue engineering, focusing on vascular bioprinting, and virtual and mixed realities leading to an overall outlook of the field and its remaining challenges.

## From Medical Images to Virtualization

**Figure 1:**
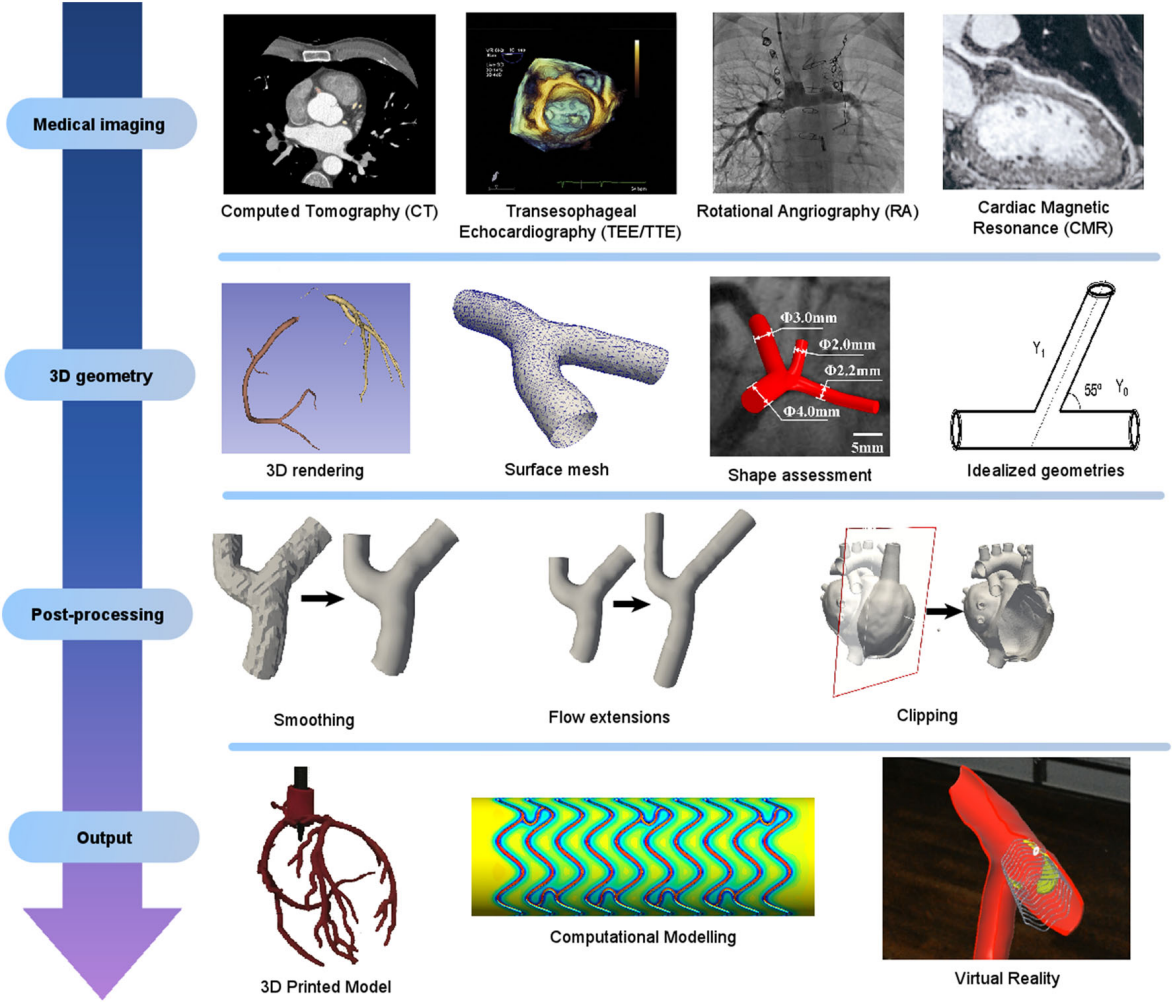
Cardiovascular 3D printing workflow. 3D printing workflow is based on the acquisition of 3D volumetric medical images from Computed Tomography (CT), TransThoracic/TransEsophageal Echocardiography (TTE/TEE), Rotational Angiography (RA) or Cardiac Magnetic Resonance (CMR). The selection of the imaging acquisition modality depends upon the cardiac structure of interest, whereby CT has generally a higher spatial resolution whilst MR has no risk of ionizing radiation. The resulting 2D image stacks are then processed into a 3D geometry through segmentation, allowing the contouring of the region of interest in the 2D image stacks before 3D rendering, mesh surface generation and possible shape assessments. Idealized geometries may also be used which relate to but do not exactly replicate underlying medical data. Post-processing then allows editing these virtual representations further through, for example, smoothing, adding of extensions, clipping and/or other operations. Once completed, direct 3D printing is possible using the standard Standard Tessellation Language (STL) file format and a range of available technologies and materials may be used complementary to virtual reality and computational modeling which use the same input format. 3D printed model^154^ partly reprinted with permission from Elsevier, TEE^86^ reprinted with permission from Elsevier, Rotational angiography^111^ with permission from Springer, CMR^127^ with permission from American College of Cardiology, Idealized model from Reference [113] with permission from Elsevier

The overall workflow for cardiovascular medical image processing is shown in Fig. 1, whereby first the cardiovascular medical images are acquired before they can be processed to 3D virtual representations and eventually used for 3D printing and complimenting outputs such as computational modeling for virtual and mixed realities.

### Cardiac Image Acquisition

The required medical imaging dataset must be volumetric, which limits cardiovascular imaging modalities to 3D echocardiography, electrocardiography-gated computed tomography (ECG-gated CT), and cardiac magnetic resonance (CMR).^55^ These have a standard format referred to as DICOM images, for Digital Imaging and Communications in Medicine.

Volumetric 3D echocardiography, either transthoracic (TTE) or transoesophageal (TEE), is a convenient modality as it is generally widely available, cost-effective and has no risk of radiation. However, as it is based on ultrasound, it is only suitable for large and clear structures, such as ventricular chambers and valve leaflets.^50^ It therefore is also subject to artefacts and anatomic data loss within the ultrasound ‘shadow’ and has a limited field of view.

For this reason, ECG-gated CT is the principal cardiovascular imaging modality today, providing sub-millimeter resolution and clear tissue structures such as calcium deposition. CT is able to image patients with pacemakers, pacemaker wires and general metal implants, making it a common imaging method before surgical or other structural interventions. It is therefore commonly used for idealized, patient-specific,^11^ and even large population studies.^96^

CMR or magnetic resonance angiography (MRA) is limited due to its comparatively lower spatial resolution (up to 1.2 mm), making it not suitable for small-scale structures such as the coronary arteries or morphological heart valve features.^169^ It is also incompatible with metallic implants,^38^, and requires sedation in 1–15% of patients due to claustrophobic reactions.^38^ Still, unlike CT, CMR does not require ionizing radiation or costly iodinated contrast media to distinguish tissue compositions.

For all of these modalities, the derived images and hence the final phantom quality depend on sufficient signal intensity and tissue contrast whilst enabling minimal image artefacts. Thus, both the cardiac movement and breathing artefacts challenge image acquisition, and compensating techniques such as gating and breath-holding may be incorporated (Table 1).

**Table 1:** Overview of recent 3D printing studies, including the imaging and technology used in the cardiovascular field.

Topic		Imaging	3D printing
Education: Patient communication, learners training, surgical simulation			
Medical training	Congenital heart anomalies^34^,^48^,^71^,^106^	CTA\CMR	MJ/BJ
	Endovascular simulation for training in guide wire and catheter based skills^85^	CTA	SLA
	Valve models for training and planning heart surgery^62^	Ultrasound	FDM mold using DragonSkin
Research: Visualisation, analysis, device testing			
Aorta	Aortic stenosis^90^ Aortic valve regurgitation^153^ Stiff aortic arch^23^,^60^,^77^,^99^	CTA CT CMR TEE	FDM SLA
Coronaries	Coronary arteries^147^ Vasculature^65^	CTA CMR	FDM SLA
Functional	Vessel compliance^15^, specifically coronary compliance^21^	CMR	MJ
	Epicardial coronary perfusion^165^	CTA	
Visualisation	Flow visualisation with PIV^167^ most notably aortic stenosis^154^	CTA	
Analysis	Fractional Flow Reserve (FFR) measurement^74^,^147^	Idealized	MJ
	*In vitro* Doppler tracing measurements of mitral valve inflow^93^	TEE	FDM mold using Moldstar and EcoFlex
	General stenotic phantoms under pulsatile flow^65^	Idealized	SLS
	Coronary stents performance^154^, including drug eluting stents^28^, and stent placement^157^	CTA	MJ
Clinical: Surgical planning, intra-operative guides			
Device testing	LAAO device testing^37^	CTA	SLS/FDM
Patient-specific optimization	LAAO transseptual puncture^31^	CT	SLA
	Surgical Planning^92^	CTA/CMR	BJ
	Congenital heart models across a range of age, pathology and imaging techniques^7^	CTA/CMR	FDM
	TAVR Assess aortic root strain^117^	CTA	MJ
	3D print modeling of congenital heart chambers^15^	CMR	MJ
	Reconstructive modeling of intracardiac tumors^41^	CTA	FDM
Clinical Software Validation	CT-based FFR analysis^143^	CT	FDM
Procedural planning	LAAO^56^	CT	FDM
	Prediction of coronary collusion following TAVR^132^	CTA	BJ
	Percutaneous structural intervention^36^	CTA	SLA
	Aortic arc obstruction^71^	CTA	MJ
	Mitral valve-in-valve interventions^2^		
	Congenital heart models^26^,^27^,^131^	CTA/CMR	BJ
Rare interventions	Transcatheter plug implantations for a mitral perforation^81^	CTA	
Devices and innovation: Bioprinting			
Large vessels	EC–SMC co-culture to mimic vascular complexity^73^ Interactions device-cells: stentable artery^5^	Idealized	FDM hydrogel
Small vessels	EC-pericyte co-culture to mimic vascular complexity^25^,^69^,^78^,^146^ Angiogenesis: studying de novo vessel formation^70^,^161^ Microvascular disease modeling^19^,^72^,^80^,^89^,^139^,^172^	Idealized	Hydrogel SLA

*BJ* binder jetting, *CMR* cardiac magnetic resonance, *CTA* computed tomography angiography, *EC* endothelial cell, *FDM* fused deposition modeling, *LAOO* left atrial appendage occlusion, *MJ* material jetting, *SLA* stereolithography, *SLS* selective laser sintering, *SMC* smooth muscle cells, *TAVR* transcatheter aortic valve replacement

### Segmentation and Virtual Reconstruction

The medial images acquired are 2D image stacks representing the 3D volume, requiring processing so that the region of interest within each of the images can be discriminated against the surrounding tissue. After, the 2D stacked contours require further reconstruction into a 3D representation.

Several cardiovascular image segmentation and reconstruction tools exist today with varying applicability, advantages and disadvantages, including open-source software such as Seg3D, 3DSlicer, InVesalius, ITK-Snap, Osirix Lite, Horos, ImageJ, Blender, Medical Imaging Interaction Toolkit (MITK), and Vascular Modeling Toolkit (VMTK) along with popular proprietary software such as Amira, Mimics (Materialise NV, Leuven, Belgium), and clinical software such as Vitrea (Vital Images, Inc.), Intuition (Tera Recon), AW Volumeshare (GE Healthcare, IL, USA) (Table 2).

**Table 2:** Overview of common medical image segmentation and virtual geometry processing software, adapted from Ref. 22

Name	Segmentation Tools	Additional Features	**License**
Seg3D^135^	$\bullet$ Manual modification	$\bullet$ Multiple segmentations possible	Free
	$\bullet$ Thresholding	$\bullet$ Intuitive layer based interface	
	$\bullet$ Edge detection (Canny edge filter)	$\bullet$ Tools available to edit images and	
	$\bullet$ Level Sets	segmentations (e.g. erosion, holefilling	
	$\bullet$ Connected component filter	and boolean combinations)	
		$\bullet$ Distance maps	
3DSlicer^42^	$\bullet$ Manual modification	$\bullet$ Image registration	Free
	$\bullet$ Thresholding	$\bullet$ Popular for 3D visualization	
	$\bullet$ Edge detection (Watershed filter)		
	$\bullet$ Fast marching method		
	$\bullet$ Grow cut method		
	$\bullet$ Level tracing method		
	$\bullet$ Range of modified filters		
InVesalius^3^	$\bullet$ Manual modification	$\bullet$ Simple interface	Free
	$\bullet$ Thresholding	$\bullet$ Automatic thresholding from CT	
		$\bullet$ Popular for 3D visualization	
ITK-Snap^171^	$\bullet$ Manual modification	$\bullet$ Simple interface	Free
	$\bullet$ Edge detection (active contour)	$\bullet$ Multiple segmentations possible	
Osirix Lite^109^	$\bullet$ Manual modification	$\bullet$ Macintosh only	Commercial (Osirix)\
Horos^58^	$\bullet$ Edge detection (region growing)	$\bullet$ For visualization and image fusion	Free (Horos)
		$\bullet$ Freeware versions of OsirixMD	
		which is certified for clinical use	
ImageJ^125^	$\bullet$ Extract mesh based on intensity	$\bullet$ 2D image processing platform	Free
	isosurface	$\bullet$3D viewer plug-in	
Mimics^94^	$\bullet$ Thresholding	$\bullet$ Multiple segmentations possible	Commercial
	$\bullet$ Region growing	$\bullet$ Integrates with 3-matic for	
	$\bullet$ Manual modification	further 3D printing processing	
AW Volumeshare^9^	Tracking	$\bullet$ Automatic labeling of coronary	Commercial
	$\bullet$ Manual modification	arteries	
MITK^163^	$\bullet$ Combines functionality of the Insight Toolkit (ITK) and	$\bullet$ Multiple, consistent views of	Free
	the Visualization Toolkit (VTK)	co-registered 3x orthogonal 2D and 3D	
		$\bullet$ Interaction, undo and redo concepts	
		$\bullet$ Repository features	
VMTK^4^	$\bullet$ 3D reconstruction	$\bullet$Centerlines	Free
	$\bullet$ Geometric analysis	$\bullet$Post-processing	
	$\bullet$ Mesh generation		
	$\bullet$ Surface data analysis		
Blender^17^	$\bullet$ 3D creation suit including modeling,	$\bullet$ Video editing	Free
	rigging, animation, simulation,	$\bullet$ 2D animation pipeline	
	rendering, motion tracking	$\bullet$ Motion tracking	

Segmentation processes can be automated using centerline and boundary extraction methods, or manually using editing, extraction and cropping, or most commonly in a semi-automated workflow whereby initial automated segmentation is followed by manual expert correction.^49^ Common semi-automated segmentation methods include brightness thresholding and region growing, dynamic region growing, active contouring, and edge detection (Watershed filter).^24^ It should be noted that many automated segmentation tools are insufficient, with advanced methods (semi-automated or manual) being required frequently to generate meaningful virtual representations from the DICOM images. Many of the available tools therefore require a high level of expertise and time commitment,^24^ and increasingly the field is trending towards more automated volume extraction methods,^100^,^120^ for example with the use of machine learning.

After relevant contours of the 2D regions of interest are marked or segmented, the perpendicular surface contours can then be reconstructed to form a surface using a number of available iso-surface extraction algorithms. Marching cubes^83^ or more modern algorithms such as flying edges^133^ are commonly used and are integrated as standard in relevant software. Specific image processing strategies have also been developed to enhance vascular structures such as the widely used Frangi vesselness filter.^43^,^44^,^101^ More advanced filters, such as Vessel Enhancing Diffusion (VED) for vessel edge and coherence enhancement,^88^ have resulted in high accuracy segmentation, although their use is not as common due to high computational costs. Implementations of these algorithms are available in most medical image processing toolkits, including VMTK. The resulting virtual representations are then stored in form of surface information, whereby a point cloud and its mesh triangulation describe the surface and its connectivity across even complex shapes.

After segmentation, the now virtual structures require further processing for mesh smoothing, editing and improving these surface representations further. Powerful open-source tools exist for this purpose, including VMTK^4^ and MeshLab.^30^ VMTK is particularly strong for various computational geometry operations relevant to processing vessels, computing centerlines and addition of flow extensions. MeshLab offers direct manipulation, cutting and refinement of triangulated meshes. The most common file format used is the Standard Tessellation Language or STL format, which can serve as a direct input to 3D printers. Computer Aided Design (CAD) tools may also process the virtual geometries further, often for geometry modifications such as extension, color coding of regions of interest, texturing blended material, or adding other components like threaded extensions for *in vitro* mock loop compatibility. A major disadvantage of these tools is the required software expertise and challenging learning curve. The combination of these steps often requires a significant amount of learning and expertise, and thus offers opportunities to increase user-friendliness of relevant software.

## 3D Printing Technologies and Materials

Once a virtual model is created, various methods can be used to print 3D phantoms. 3D printing with a range of materials including polymers, metals or alloys has been used since the 1970s and has become a well established technology, which is increasingly affordable, convenient, and accurate in resolution and complexity.

Initially, phantoms were produced by conventional methods such as casting and molding. This approach is predominantly injection molding-based where a large range of materials are available including PMDS, MoldStar 15, EcoFlex 00-30 and DragonSkin. Such materials including PDMS (poly(dimethylsiloxane)) SYLGARD elastomers often involve individualized or patient-specific phantoms. These models can be transparent, which offers opportunities for a range of functional flow assessments including Particle Image Velocimetry (PIV).^167^ MoldStar has the advantage of bonding to Ecoflex and DragonSkin to fix boundaries or locally ridgify structures^91^ where the Ecoflex series has the ability to undergo large deformation^91^ and DragonSkin has among the highest tensile strength.^91^ MoldStar 15 and EcoFlex 00-30 were used as negative and positive molds for mitral valve *in vitro* experiments respectively,^93^ and DragonSkin has been successfully applied for heart surgeon training and planning.^62^ Disadvantages of casting and molding include high cost of singular cast creation, time-consuming preparation steps, and limited resolution. Consequently, previous research often mass-produced population-averaged and idealized models which may limit the relevance of the obtained results and observations.^51^

**Figure 2:**
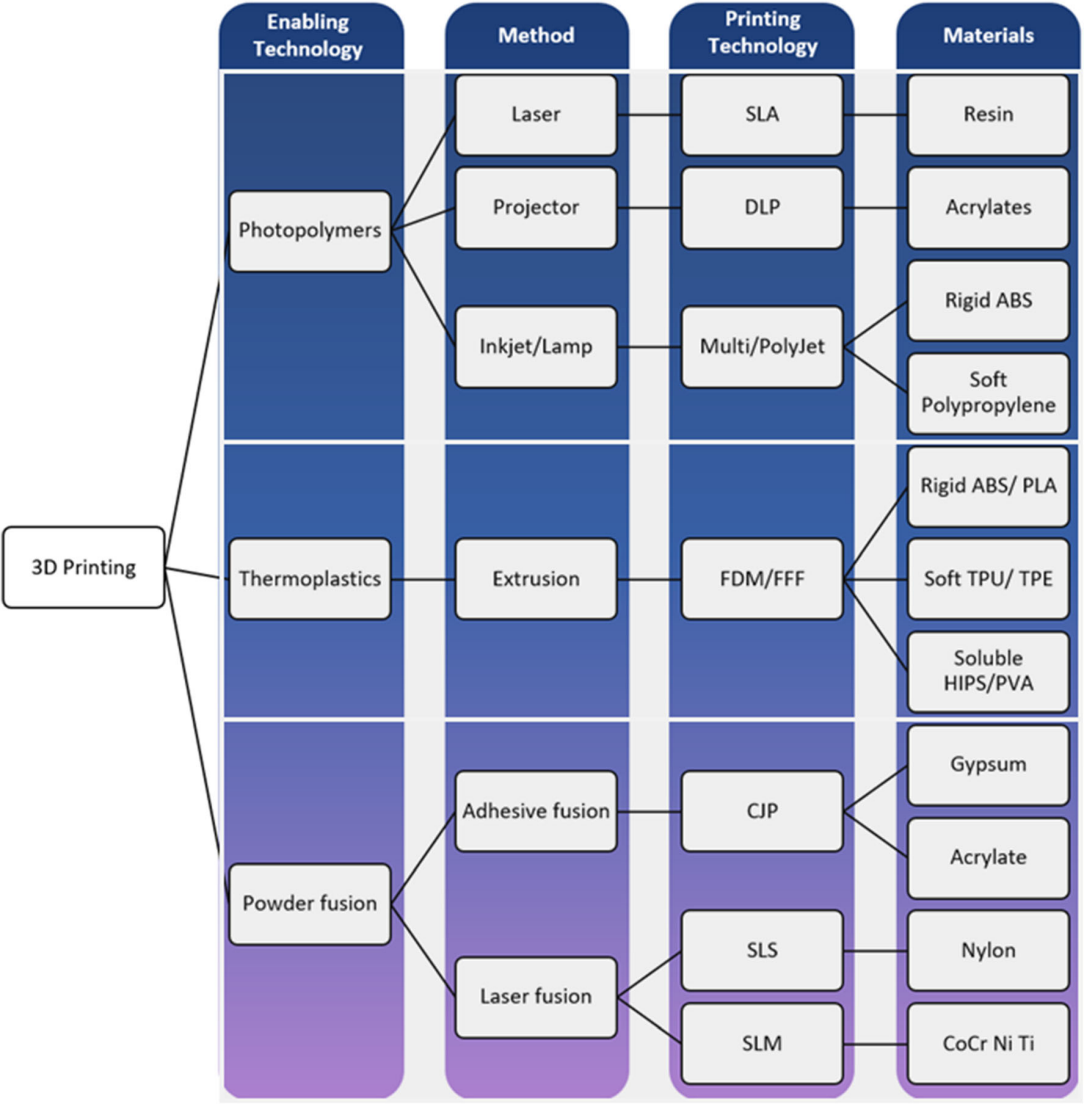
Overview of 3D printing technologies, adapted from Ref. 18. Abbreviations as follows: *FDM* fused deposition modeling, *SLA* stereolithography, *DLP* digital light processing, *ABS* acrylonitrile–butadiene–styrene, *PLA* polylactic acid, *TPU* thermoplastic polyurethane, *TPE* thermoplastic elastomers, *HIPS* high impact polystyrene, *PVA* polyvinyl alcohol, *CJP* colour jet printing, *SLS* selective laser sintering, *SLM* selective laser melting, *CoCr* cobalt–chromium, *Ni* nickel, *Ti* titanium

Unlike casting or molding, 3D printing is fundamentally a layer-by-layer build-up technique that relies on solidification of the material between each layer. 3D printing technologies can overcome some or all of the drawbacks of casting and molding, offering patient-specific, fast and precise production of phantoms at a low cost by eliminating the need for tooling. Various 3D printing technologies are available today and an overview is provided in Fig. 2.

**Figure 3:**
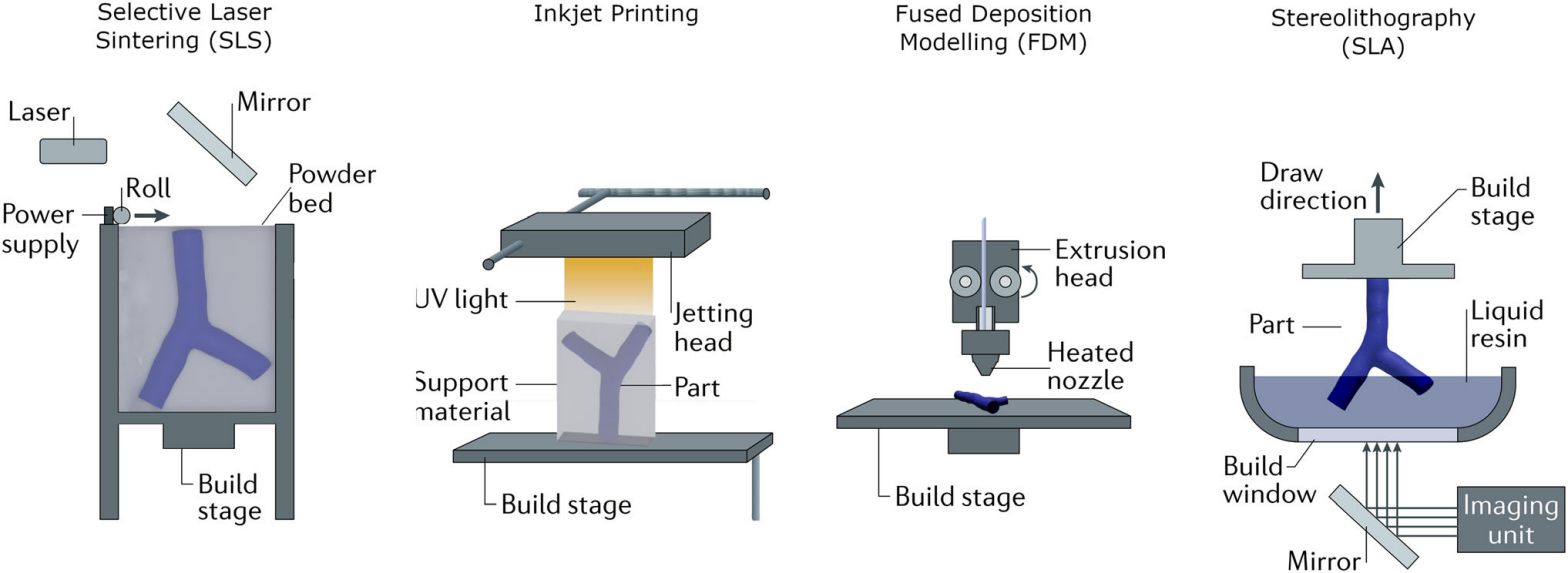
Commonly used 3D printing technologies, adapted from Ref. 156

3D printing for cardiovascular applications is challenging, and suitable methods should be selected depending upon many factors including resolution, materials, the required physical properties such as complexity, color/transparency, durability, biocompatibility, cost and recyclability. Similarly, post-printing requirements are an important consideration especially for some anatomies (such as vessels), which may include cleaning, removal of support materials, ultraviolet curing, sterilization and labelling.^49^ A range of technologies and materials are suitable for cardiovascular 3D printing (Fig. 2), and the most frequently used include Stereolithography or ‘SLA’, material jetting ‘MJ’, Fused Deposition Modeling ‘FDM’ (material extrusion), and Selective Laser Sintering ‘SLS’ (Fig. 3).^18^ These can be categorized according to their technology used: photopolymerization (SLA and MJ), thermoplastic (FDM), and powder fusion (SLS) as detailed below and compared in Table 3.

**Table 3:** Cardiovascular 3D printing technologies, their accuracy, cost, advantages and disadvantages, adapted from Refs. 22, 40, 120, 156

Printing techniques	Accuracy	Cost	Advantages	Disadvantages
Photopolymerization: stereolithography (SLA)	1–50 *μ*m	$$	$$\bullet$$ Moderate cost	$$\bullet$$ Prints are prone to slight distortions
	***		$$\bullet$$ Good surface finish	$$\bullet$$ Curing resins need to be handled with care
			$$\bullet$$ High resolution	$$\bullet$$ Moderate strength
			$$\bullet$$ Large part size	
Photopolymerization: material Jetting (MJ)	50 $$\mu$$m	$$	$$\bullet$$ Very good surface finish	
	**		$$\bullet$$ High resolution	$$\bullet$$ High material cost
			$$\bullet$$ Ability to gradually combine	$$\bullet$$ Curing resins need to be handled with care
			different polymers	
Thermoplastic technology$$\backslash$$extrusion printing: filament deposition modeling (FDM)	100 $$\mu$$m	$	$$\bullet$$ Low material costs	$$\bullet$$ Rippled and porous surface
	*		$$\bullet$$ Simple to use	$$\bullet$$ Fragile along *Z*-axis
			$$\bullet$$ Low cost printers available	$$\bullet$$ Low Speed
			$$\bullet$$ Good strength	
Powder binding: binder jetting (BJ)	100 $$\mu$$m	$$	$$\bullet$$ Can include colour	
	*		$$\bullet$$ Quick	$$\bullet$$ Printers are expensive
			$$\bullet$$ Low material costs	$$\bullet$$ Rough Surface finish
			$$\bullet$$ Many materials available	
Powder binding: selective laser sintering (SLS)	100 $$\mu$$m	$$$	$$\bullet$$ Prints are strong	
	*		$$\bullet$$ Many materials available	$$\bullet$$ Printers are expensive
			$$\bullet$$ Large part size	$$\bullet$$ Rough and powdery surface finish

### Photopolymer Technology

SLA uses photopolymeric resin which is solidified using a digitally guided ultraviolet laser (or sometimes a visible light source). It can achieve smallest minimum feature sizes and very high resolution, however it incurs a high cost and has a limited ability for printing complex features as often required for cardiovascular research purposes. It is capable of printing translucent prints relevant for some functional modeling.^41^

MJ for PolyJet/MultiJet or Material Jetting refers to processes similar to SLA with orifice jetting of both a photopolymer for the actual model (solidifies through light exposure as before) and a photo-curable gel (PolyJet$$^{\mathrm{TM}}$$ trademarked by Stratasys) or wax (used by 3D Systems) as removable support material. This technique enables diverse and complex features, multiple materials and even colors to be printed simultaneously. It is thus capable of highly complex models with thin walls and smooth surface finishes equivalent to SLA with up to 160 $$\mu$$m resolution, making it a favorite tool for multi-material prints.^61^ A major drawback of this technology is its high printer and material cost, ranging from $50k to 500k USD and $300 USD/kg respectively.^41^

A popular material called TangoPlus has previously been used to manufacture arterial phantoms,^15^ allowing inexpensive and rapid fabrication of non-uniform wall thicknesses due to its PolyJet compatibility. TangoPlus outperformed cast PDMS,^32^ being compliant and thus suitable for mock-loop *in vitro* testing and pre-operative mock device insertion.^15^ Still, the material exhibits unrealistic isotropic behavior and has been reported to be too stiff to resemble either compliant systemic venous systems^15^ or the low bending modulus of healthy porcine mitral valve tissue.^155^ It should be considered, that while TangoPlus is superior to cast PDMS, other casting material such as the combination of MoldStar 15 and EchoFlex may outperform TangoPlus in these aspects which has not yet been explored, and thus warrants future studies.

Patient-specific vascular phantoms from PolyJet printers were found to be highly accurate with $<125$ $\mu$m surface differences,^63^ rendering them suitable for device testing and general research.^63^ Cleaning the support material remains challenging for tortuous and small vessels of less than 2 mm diameter, and it is recommended to print such small vascular structures upright.

### Thermoplastic Technology

FDM extrudes melted thermoplastic filaments layer-by-layer together with a support material, which is later dissolved. While FDM produces less fine feature sizes, it is substantially lower in cost in terms of materials and the printer itself. Its output may be suitable for many applications in the cardiovascular filed including pre-surgical applications.^41^

### Powder Fusion Technology

BJ is also referred to as Inkjet 3D printing and uses two materials to build objects: a powder-based material—usually gypsum, and a bonding agent to create an adhesive bond between the powder layers. Common BJ materials include ceramics, metals, sand and plastics. SLS uses a high-power laser beam to create strong parts of fused metal or ceramic powder and is preferred for building functional prototypes such as mitral valve models.^16^ The capability of printing in color is a major benefit resulting in multiple applications whereby, for example, the anatomy of arteries and veins, congenital defects, infarct regions, or any other area of interest can be highlighted.^41^

## Milestone Applications of 3D Cardiovascular Printing

**Figure 4:**
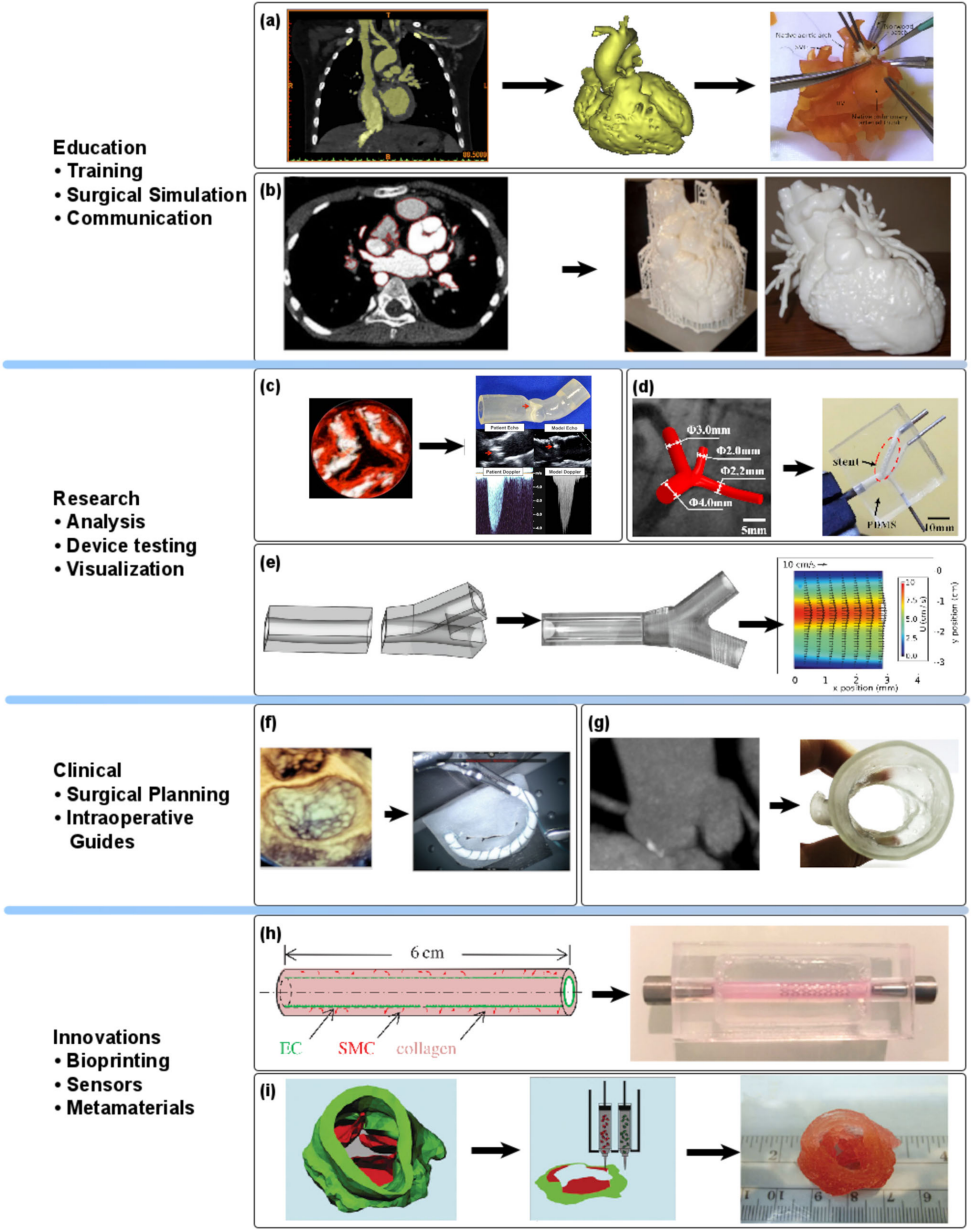
Key studies: **a** 3D printed model of patient with tetralogy of Fallot used for hands-on surgical training.^168^**b** 3D model of the heart used to explain congenital heart disease to patient and his parents.^14^**c** Stenosis flow analysis^90^ using several patient-specific models. Doppler flow analysis of the resulting models shows close agreement with patients’ velocity and ejection time. **d** PDMS model of coronary arteries used to test stenting strategies and validate computational simulations of stent placement.^157^**e** 3D printed model of inferior vena cava,^10^ demonstrating feasibility of printing transparent models with index of refraction matched to the fluid used to allow Particle Image Velocimetry (PIV) measurements without distortions. **f** 3D printed phantom of the mitral valve used for surgical planning and training.^116^**g** Aortic root model transcatheter aortic valve replacement planning. Models showed great agreement with the physiological measurements and could be used to predict Paraaortic regurgitation with reasonable accuracy.^122^**h** Stent implantation in bioprinted artery, including smooth muscle cells and endothelial cells.^5^ Flow disruption due to the stent is measured using PIV and showed close agreement with computational models. Endothelial cell healing and smooth muscle cell migration post-stenting to demonstrate feasibility of measuring biological response. **i** 3D printed Aortic valve conduits,^39^ incorporating smooth muscle cells and valve leaflet interstitial cells within alginate/gelatin hydrogel. The resulting model had good fidelity and incorporated cells remained viable, potentially providing a method of valve replacement with biologically active material. Panel **a** reprinted with permission from Elsevier, Panel **b** and **d** under Creative Commons license, Panel **c** with permission from Wolters Kluwer Health, Panels **e** and **g** with permission from Springer, Panel **h** with permission from Royal Society, Panel **i** with permission from Wiley Periodicals

In recent years, increased image resolution and advanced non-invasive techniques have transformed the field of medical imaging towards increasingly informative radiology diagnostics, forming the basis for advanced 3D printing methodologies and in turn increasingly realistic anatomical phantoms with pivotal impact on cardiovascular education, research and its clinical translation. A range of applications derive from physical models in cardiovascular sciences as shown in Fig. 4. An overview of such applications is provided by Sun *et al.*,^144^ and especially for diseased anatomy in Giannopoulos *et al.*,^49^ with both reviews having a clinical focus. Here, we review the latest literature regarding applications in education, research, and clinical translation before outlining the fields overall development including competing and complimentary technologies and trends.

### Educational Tools

Traditionally, medical knowledge is taught by the use of human cadavers in medical school, while later junior doctors shadow more senior colleagues and scrub for surgeries. Having visualization beyond a 2D flat screen can therefore greatly assist, especially for challenging anatomical and pathological conditions.^115^ Instructional models of normal and abnormal structural relationships are available using plastic heart models, allowing complex anatomical arrangements with the promise to transform medical education.^150^ Several simulation based training tools have been developed to aid the training of surgeons^53^,^119^,^121^ using animal tissue or other synthetic approaches, enabling the improvement of surgical skills through better tactile and anatomic understanding. In fact, it was shown that clinicians felt 3D printing was a invaluable addition to common imaging and 3D rendering techniques,^16^ with the advantage of being more flexible and able to capture fine anatomical details compared to tools mentioned above.

In recent years, patient-specific anatomical models especially of pathology have been more widely accessible due to the rapid improvement of 3D printing technology.^46^ This has proven widely beneficial, especially for children and adults with congenital heart disease, whose anatomy is often unique after rare congenital defects are repaired via multiple surgical interventions.^140^ Patient-specific models of congenital heart anomalies have been utilized for training and communication in multiple studies.^48^,^71^,^106^

3D models can also enhance learning of inexperienced trainees. The growth in adult congenital and structural heart interventions and their associated technologies lends itself particularly well to being taught through 3D modeling for training with novel devices such as in mitral valve interventions and left atrial appendage occlusion. Indeed, some early studies have already demonstrated the application of 3D printed models for endovascular simulation for training in guidewire and catheter-based skills.^85^

### Research Applications on Functional Replications

Standard 3D printing for cardiovascular research mainly evolves around the manufacturing of functional models, most notably ones made from transparent materials to allow flow quantification techniques such as PIV for direct visualization of complex flow dynamics.^167^ Such haemodynamic flow conditions are frequently stimulated by Mock Circulatory Systems (MCS). These may be mechanical, hydraulic and electric systems which are designed to facilitate *in vitro* testing for in depth investigation into the dynamics of the cardiovascular system.^138^ Developments in the construction of MCSs have led to a substantial increase in the variety of configurations available, broadly extending the range of experimental study. By characterizing the system into three separate subsystems, namely (i) motion and driving, (ii) fluid, and (iii) measurement.^138^ Modifications to each subsection can act to focus the investigation. Advancements in the motion and driving subsystem has enabled both steady state or pulsatile flow to be used, whereby a pulsatile flow setup was previously used to closely mimic physiological blood flow fluctuations of the cardiac cycle.^138^ Incorporation of different sized valves and compliance chambers may replicate physiological impedance, and the choice of fluid used offers control over shear-thinning and thickening properties^12^ as a means of evaluating the performance of various functional models even on different scales. Example applications include total artificial hearts, aortic and mitral valves, and blood vessels.^138^ The performance of stents,^154^ including Drug Eluting Stents (DES),^28^ has also been successfully evaluated *in vitro* before. Analysis of stent placement was also accomplished using 3D printed surgery models.^157^

Vessel compliance has been accounted for using PolyJet printing with TangoPlus,^15^ with compliant coronary models having been tested.^21^ Most research to date used rigid aortic arch models.^23^,^60^,^99^

Idealized stenotic phantoms were used to measure flow under pulsatile conditions,^65^ and common cardiac pathologies such as aortic stenosis have been studied, with implications for clinical scenarios such as “low flow-low gradient”.^154^ A patient-specific aortic valve regurgitation model showed close agreement with respective clinical Doppler measurements.^153^

Together, these studies generate further understanding of physiological and patho-physiological mechanisms today and may underpin studies for the preparation and/or development of interventional procedures in the future.

### Clinical Translation

Open surgical, percutaneous and transcatheter cardiac procedures require a thorough knowledge of human anatomy and topographical relations of various anatomical structures. The possibility of having a physical 3D model goes beyond closer inspection with kinestatic learning and actually facilitates the practice of surgical procedures^87^ resulting in a number of impactful studies in this field.

Left atrial appendage occlusion (LAAO) has thus far been the procedure most intuitively suited to 3D printing from both a device and procedural planning perspective due to the variability in patient anatomy and thus the importance of appropriate device selection and sizing. With 3D printing *in vitro* testing, minimal leakage and no perforation was achieved,^37^ and critical LAAO procedural steps have been personalized based on a 3D model-guided approach,^31^ with prospective trials confirming its feasibility and validity.^56^

Qian *et al.*^117^ assessed patient-specific aortic root strain *in vitro* after trans-catheter aortic valve replacement (TAVR), a widely performed structural heart intervention. Other patient-specific risks of complications could potentially be minimized or even eliminated using 3D printed procedural planning, with coronary occlusion being retrospectively predicted using a 3D TAVR case model following a fatal complication.^132^ Especially rare, high risk cases such as the so called “valve-in-valve” procedures may benefit greatly from such 3D print derived pre-procedural insights.

A full 3D heart model was used for percutaneous structural intervention,^36^ and the pre-surgical planning of an aortic arch obstruction was also previously accomplished.^71^ Similarly, mitral valve-in-valve interventions carry a significant risk of left ventricular outflow tract obstruction, and preprocedural 3D planning may be an avenue for accurately predicting the associated risk and other complications such as device embolization.^2^ Rarer valve interventions, such as transcatheter plug implantations for a mitral perforation have also been described in the literature.^81^ More broadly, the application of transcatheter mitral valve repair technology in general may benefit from improved procedural planning with patient-specific features such as where and how to grasp leaflets during a MitraClip procedure.

Interventional pre-operative planning and simulations through congenital heart models have been accomplished in scenarios ranging from double outlet right ventricle,^170^ atrial and ventricular septal defects,^27^,^131^ Tetralogy of Fallot,^126^ and hypoplastic left heart syndrome and aortopathies,^26^ even across a range of age, pathology and imaging techniques.^7^

Comparatively, the “bench-testing”, for example of coronary artery stenting has been less developed due to the use of idealized models which do not incorporate disease and use a single material,^108^ which is not indicative of complex patterns of atherosclerotic disease. There is particular interest in the potential for 3D printing of coronary bifurcation anatomy and disease—a commonly encountered lesion sub-set for interventional cardiologists and associated with higher adverse clinical event rates. Theoretically, 3D printing of bifurcation disease anatomy specific to a given patient may permit acute assessment of interventional stent strategies and thus preplanned and personalized therapy.

In all these scenarios, the use of such technology in procedural planning may result in reduced operating time and thus may lead to more effective cost, time and resource management and ultimately may improve treatment success rates.^8^

## Future Technologies and Innovations

### Towards Realistic Material Behavior

**Figure 5:**
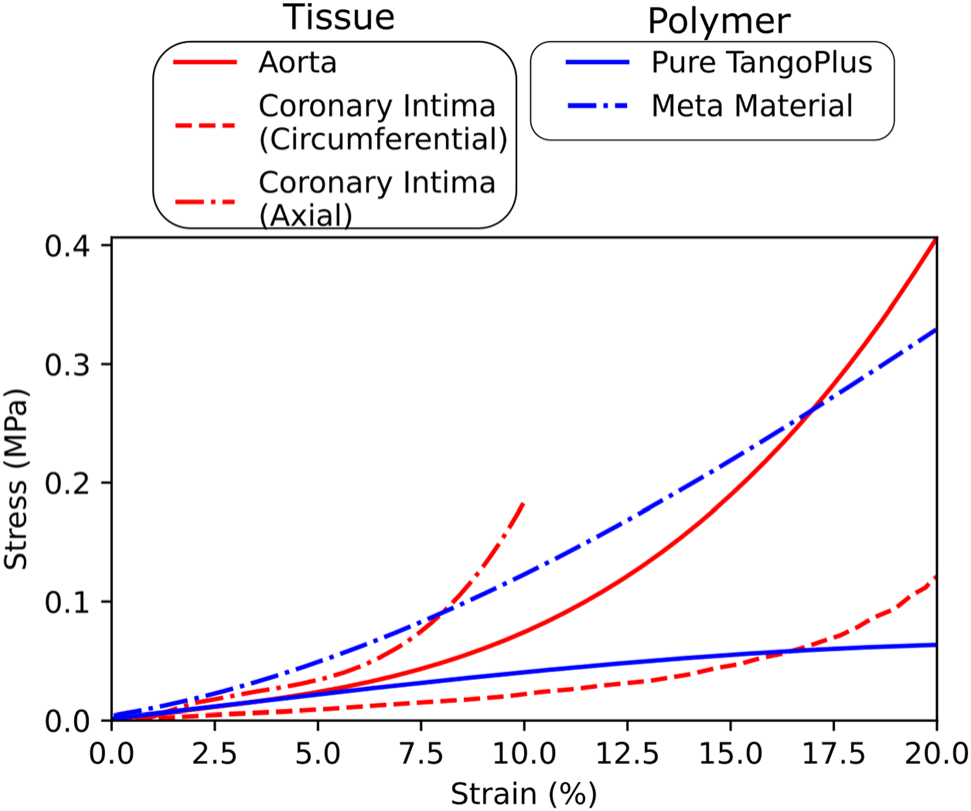
Comparison of the mechanical behaviors of aortic and coronary tissue as example for soft tissue (red) and two examples of polymer of a pure TangoPlus phantom and a meta-material phantom (blue) are shown. The strain range corresponds to the strain range used in modeling physiological tissue. Aorta and polymer stress and strain data gathered from Reference [159], Coronary Intima mechanical properties gathered from Ref. 57

Most medical phantoms are fabricated from polymeric materials with uniform tensile properties and with similar behaviors to human soft tissue when under small strain ($< 3$%). However, polymers show significantly different behavior from human soft tissue under larger strain due to due to the initial strain-stiffening behavior of soft tissue. This leads to a significant difference in the behavior of soft tissues compared to polymers under larger deformation.^118^ Despite the longer working strain of soft tissues, their behavior exceeding the dynamic strain range is largely unknown. Further research on the full extent of the stress–strain relationship is needed to better understand the behavior of the cardiac tissue as it exceeds the working strain range. Polymers, on the other hand, behave close to linear with a much lower stiffening compared to real tissue. This means that even though the initial Young’s modulus can be matched, their mechanical behavior overall deviates substantially from native tissue (Fig. 5a Dynamic strain range). Additionally, not all human cardiovascular tissue has been quantified in its mechanical behavior, limited to the healthy and diseased coronary arteries^66^ and aortic arch.^47^ Additionally, animal studies have also indicated the complexity of behavior between differing blood vessels throughout the body, concluding that the mechanical properties of a blood vessel depend not only on the inherent characteristics of the vessel wall but also on the behavior of surrounding tissues and vessels.^45^ Real tissue’s directional structures, and science’s inadequacy in matching these to date, is therefore a major drawback in all phantom studies,^63^ and thus limits 3D printed studies’ efficacy for the translation into clinical practice.^15^

One study attempted to embed fiber-like micro-structures with different materials and shapes within the usually heterogeneous phantoms to create the first tissue-mimicking phantom,^158^ and matched aortic tissue^159^ for TAVR planning^117^ (Fig. 5c). This concept of “metamaterials” refers to secondary material structures within the prints, whereby the overall behavior derives from both the properties of the constituting materials and from their geometrical arrangement^79^ and represents a major advance in the field of benchtop testing with phantoms.

### Bioprinting and Tissue Engineering

Unlike traditional 3D printing, 3D bioprinting or biofabrication is an extension of traditional fabrication approaches where the product is a biological material such as extracellular matrix (ECM) proteins or even living cells. Whilst cardiovascular 3D bioprinting and molecular 3D printing hold revolutionary potential, they are currently not translated into clinical practice. Many challenges remain in the field of biofabrication of vascular tissues, and some of these challenges have been highlighted elsewhere.^103^

The ultimate goal of cellularized fabricated constructs is to capture the physiology of the complex native vasculature and to enable the investigation of living processes such as basic cellular function, vessel remodeling, or even pathological pathways. The application of 3D biofabrication to the vasculature has recently been reviewed.^102^,^128^

Biofabrication and vascular tissue engineering pose specific challenges, including dimensions and scaling, that constrain the possible technical approaches used in their production. For example, the vasculature is a hierarchical tree of vessels that spans a broad range of dimensions from several centimeters in the case of large blood vessels to a few micrometers for capillaries. Therefore, spatial control at both the macro- and microscopic scales is needed during biofabrication. Additionally, the cardiovascular tree is a large but sparse network and is thus different from small dense organs, making it harder to fabricate as a bulk material. Furthermore, the vascular wall is a heterogeneous structure that consists of a complex mix of ECM proteins as a fibrous scaffold, specialized cells with specific spatial localization, and, in the case of larger vessels, intervening structures such as the internal and external elastic laminae. Additionally, in large arteries, the outer layers of the wall have their own complex vascular network, the vaso vasora. Two additional critical constraints are cell viability and sufficiently fine spatial control. Most of the traditional fabrication technologies introduced before have been adapted for biological fabrication, aiming to meet these constraints.^112^ Main biofabrication technologies include (1) 3D bioprinting similar to traditional printing using biological rather than synthetic materials referred to as bioink,^136^ and several reviews address its ongoing research in greater detail.^110^,^129^,^130^ Bioinks are often in the form of cell-encapsulating hydrogels, natural or synthetic polymers that mimicks the native ECM. Natural materials have been favored in the recent years most notably fibrin, collagen, gelatin or alginate.^149^ The most common cell lines found in cardiovascular applications are endothelial cells, fibroblasts and smooth muscle cells, which can be derived either from primary cell lines, immortalized cell lines or stem cells.^35^ The use of patient derived induced pluripotent stem cells is particularly interesting as it allows patient-specific vascular tissue engineering. (2) Casting and molding is a simpler and more cost effective manufacturing approach whereby liquid biomaterial such as collagen or fibrin hydrogel is cast into a mold around a cylindrical rod to form the vascular lumen, which then allows for cell seeding on the formed lumenal wall and within the hydrogel itself to simulate mural cells. This has yielded excellent work of complex vessel geometries,^98^ replicated aneurysms, stenosis and bifurcations^89^ and stentable *in vitro* arteries^5^ with endothelial cell lining. (3) Microfabrication uses standard micro-patterning (photolithography) and micro-fluidics^114^ again with incorporation of biomaterials. (4) Guided self organization uses natural biological development processes to develop 3D constructs.^54^ Bioprinting as a vascular biofabrication method has made most advances compared to other methods introduced, with several recent bioprinting reports, for example for the study of thrombus formation and thrombolysis,^172^ printing of agarose template fibers inside synthetic polymers allowing generation of a network of endothelial cell-lined microvessels as small as 250 $\mu$m in diameter,^13^ fabrication of a large vascularised tissue with co-culture of endothelial cells, tissue-specific cells, and ECM components,^73^ inkjet printing of a 3D vascular network with bifurcations as well as tortuous vessels,^29^,^166^ and endothelial cell-lined microvascular networks that follow a large-scale pattern.^67^,^164^ Bioprinting can also be used for device testing, such as *in vitro* stent deployment in an artery containing endothelial and smooth muscle cells, allowing cellular responses to the stenting procedure to be monitored at high resolution in real time.^5^ PIV was used to monitor endothelial wound healing post stenting implanted in an arterial wall bioprinted using type I collagen hydrogel.^6^

Beyond research applications, the ultimate goal of biofabrication is to produce constructs with translational potential in order to improve patients’ lives. The need to implant a tissue in a patient imposes significant demands on the fabrication process in terms of durability, robustness, and integration into surrounding tissues. Once implanted, artificial vessels will be subjected to prominent environmental cues that can induce remodeling and can possibly accelerate the deterioration of the construct. Therefore, engineered vessels need to be able to not only withstand but also adapt to the dynamic chemical and mechanical environment in which they will reside. Finally, to allow transplantation, immunological matching is necessary: using patient-derived induced pluripotent stem cells (iPSCs) promises to ensure this biocompatibility, and several recent studies have focused on using these cells. More broadly, iPSCs are also very useful for research applications as they allow the study of patient-to-patient variability as well as exploration of the mechanisms governing genetic diseases.

To overcome the challenges outlined above, many technical innovations have recently emerged. We review here the most promising such innovations. The use of organoids as building blocks in the bio-ink increases cell density to better match *in vivo* values and permits the quick fabrication of larger structures.^64^,^105^ Building on this, modifying the material properties towards so-called self-healing gels allows the subsequent printing of a vascular network inside a bulk construct.^95^ In this technique, a condensed solution of organoids is poured into a mold after which a nozzle moves through the liquid condensate of organoids to print a bilayered vascular network. Finally, the bulk material is polymerized, and the lumens of the vessels are generated via dissolution of a sacrificial material. This is a significant step toward higher complexity and vascularization of bulk tissues. One study used this approach to generate a beating muscle of several centimeters with iPSCs.^142^

In all of these novel approaches, the question of perfusion of large engineered tissues will likely be front and center. This perfusion is essential not only for ensuring the long-term viability of the tissue but also for providing the relevant mechanical forces which constitute important cues for remodeling and stabilization of immature vessels.^64^

A field that will undoubtedly receive significant attention in tissue engineering in the coming years is the use of embedded sensors that will continuously monitor the structural and functional state of the engineered tissue. As in the rest of society, this push towards “smart” engineered vessels holds the tantalizing promise of providing highly personalized monitoring and tremendous amounts of data that can be mined using modern artificial intelligence algorithms. The push towards smart and communicating implantable vascular constructs raises considerable privacy concerns and ethical considerations that need to be addressed before such an approach becomes widely adopted.

Whilst these novel virtual and mixed reality technologies may approximate the utility of 3D printing in education, the tangible nature of 3D printing is both intuitive and naturalistic. No training is required to hold and rotate a printed model and, as 3D printing is a more accessible technology today, it may sufficiently fulfill the main educational goals of tactile and anatomical understanding.

### Complimentary Technologies: Virtual and Mixed Reality Experiences

Beyond 3D printing and physical replicas, new technologies are emerging, whereby a virtual experience is created to replace physical phantoms for training and preoperative planning. Whilst this technology is very much in its early stages for incorporation into standard clinical use, some steps towards such vision have been made in the cardiovascular context.

Two of the key technologies revolutionizing this domain are Virtual Reality (VR) and mixed reality experiences. VR is a fully-immersive experience that simulates virtual experiences by controlling the user’s complete visual and auditory senses.^141^ MR integrates the user’s real-world environment with virtual objects that interact with the physical elements.^20^

For the cardiovascular field, a VR simulator was developed to train intervention,^75^ whereby the interaction with a catheter was converted into a simulated surgical experience. Such VR training simulators are yet to be used during surgical procedures, however they are widely accepted for their training potential for surgical skills.^59^ A successful implementation^148^ used immersive VR to display complex congenital heart anomalies to medical trainees whereby the objects could slice, rotate and scale. A similar application was deployed in mixed reality^162^ with the use of holographic bodies to replace learning in dissection laboratories.

Alongside medical training, preoperative preparation with holographic models may be 1 day as effective as physical models.^20^ Preparation steps remain the same such as imaging, segmentation, processing, yet the need for actual printers and materials is replaced by reusable headsets, making VR holograms possibly more cost-efficient and sustainable than 3D printing long-term.^107^ However, within this advantage lies also their main disadvantage, their lack in tactility,^20^ yet latest Electrical Muscle Stimulation, or EMS, efforts aim to overcome the former drawback by providing a sense of touch.^82^

The improvement of visual representations of patient CT or MRI scan data used in VR and mixed reality simulations is also being explored with cinematic rendering as opposed to the traditional volume rendering technique used for post-processing. Cinematic rendering produces photo-realistic images and was found to provide greater depth perception and spatial impression in comparison to volume rendering, however at the cost of requiring significantly greater computing power.^123^ A study further explored the use of cinematic rendering for cardiac intraluminal visualization through a modified preset called Black Blood Cinematic Rendering, clearly identifying factors such as thrombosis and medical devices such as stents, when compared with conventional imaging.^124^

Ultimately, mixed reality technology may replace fluoroscopy during endovascular interventions like stenting and thus eliminate radiation exposure to the patient and surgical staff.^76^ This may be achieved through a catheter system with magnetic tracking, creating a ‘virtual angioscope’ once current alignment errors are overcome.

A further application incorporates the recent advancement of the digital twin in cardiovascular medicine.^33^ Integration of the digital twins with VR and mixed reality will further enhance the learning and preoperative planning capabilities. Visualizing the effects of simulated surgery on these models in 3D space will allow clinicians to evaluate the optimal computed result before the clinical procedure.

## Conclusions

Whilst 3D printing is not a new industry, it is a multi-versatile tool which offers widespread impact across many different cardiovascular fields. This review gives a unique perspective on how 3D printing technologies can and will bridge the gap between engineering tools and the future of cardiovascular care.

We outlined 3D printing workflows and elaborated on each step from image acquisition to processing and virtualization before introducing 3D printing technologies across non-biological and biological approaches. We then highlighted state-of-the-art work in the cardiovascular field from recent years, ranging from research, to clinical and educational efforts, pushing the boundaries of knowledge and know-how. Promising trends, complimentary technologies and future applications were also discussed in this context, providing a general outlook of the field’s directions over the next decade.

Overall, 3D non-biological printing is likely to continue to rapidly penetrate *in vitro* research efforts, clinical practice and education with a global push for open source virtual anatomy libraries, increasingly user-friendly image processing tools and the drive for high-precision printers to be integrated into practice with economic demands of cost and physical space. Together with emerging efforts towards more realistic mechanical behavior, this offers many opportunities for rapid on-site personalized surgery planning, promises to minimize animal testing of novel cardiovascular devices in the future and may become a new standard as a readily available educational tool.

Cardiovascular 3D biofabrication and tissue engineering remain comparatively more removed from clinical translation today but promise pivotal impact in the coming years by combining advanced manufacturing, cell biology, molecular biomarkers, and materials sciences. The most immediate impact is likely to be in the emerging field of vessels-on-chip for toxicity testing of cardiovascular drugs together with cellular response monitoring. Although the tantalizing prospect of whole organ printing and implantation into patients remains some time away, these approaches are already proving helpful by providing unique insight into biological mechanisms under controlled conditions, therefore yielding a platform for limited and initial device testing today.

Complimenting virtual and mixed reality technologies may replace the need for some 3D printing in the future, including demonstration, education and surgical planning, especially once tactile feedback has been optimized, likely forming the pathway of assisted surgery in future. This may offer some benefits in terms of 3D printing associated material cost, increase sustainability and offer additional in-depth experiences such as anatomy virtual fly-through and scaling. Potentially it could replace 3D printing for some aspects of medical planning, education and communication purposes in the near future. However, it is not capable of replacing device testing platforms and means of functional research. Ultimately 3D printing has and will continue to significantly improve training, research and communication in the cardiovascular field with related emerging technologies increasingly taking-up complimentary functions. This will elevate the rapid development towards total patient-specific cardiovascular care in the near and far future, assisting the accomplishment of previously unmet clinical outcome success rates.

## Abbreviations

2D: Two dimensional
3D: Three dimensional
ABS: Acrylonitrile–butadiene–styrene
AM: Additive manufacturing
CAD: Computer Aided Drawing
CFD: Computational fluid dynamics
CJP: Colour Jet Printing
CMR: Cardiac magnetic resonance
CoCr: CobaltChromium
CT: Computed tomography
DES: Drug eluting stent
DICOM: Digital Imaging and Communications in Medicine
DLP: Digital Light Processing
ECM: Extracellular matrix
EMS: Electrical Muscle Stimulation
FDM: Fused deposition modeling
HIPS: High Impact Polystyrene
iPSC: Induced pluripotent stem cells
LAAO: Left atrial appendage occlusion
MCS: Mock Circulatory System
MR: Mixed reality
MRA: Magnetic resonance angiography
Ni: Nickel
PDMS: (Poly(dimethylsiloxane)) SYLGARD elastomers
pH: Power of hydrogen
PIV: Particle image velocimetry
PLA: Polylactic acid
PVA: Polyvinyl Alcohol
SLA: Stereolithography
SLM: Selective Laser Melting
SLS: Selective Laser Sintering
STL: Standard tessellation language
TAVR: Transcatheter aortic valve replacement
TEE: Transoesophageal volumetric 3D echocardiography
Ti: Titanium
TPE: Thermoplastic elastomers
TPU: Thermoplastic polyurethane
TTE: Transthoracic volumetric 3D echocardiography
VED: Vessel Enhancing Diffusion
VMTK: Vascular Modeling Toolkit
VR: Virtual reality
CR: Cinematic Rendering
VRT: Volume Rendering Technique
BBCR: Black Blood Cinematic Rendering
