# Correction to: 3D Printing for Cardiovascular Applications: From End-to-End Processes to Emerging Developments

**DOI:** 10.1007/s10439-021-02894-w

**Published:** 2022-01-20

**Authors:** Ramtin Gharleghi, Claire A. Dessalles, Ronil Lal, Sinead McCraith, Kiran Sarathy, Nigel Jepson, James Otton, Abdul I. Barakat, Susann Beier

**Affiliations:** 1 Faculty of Engineering, School of Mechanical and Manufacturing, UNSW, Sydney, Australia; 2 LadHyX - UMR 7646, École Polytechnique, Palaiseau, France; 3 Prince of Wales Hospital, Sydney, Australia; 4 Prince of Wales Clinical School of Medicine, UNSW, Sydney, Australia; 5 Department of Cardiology, Liverpool Hospital, Sydney, Australia

## Correction to: Annals of Biomedical Engineering, Vol. 49, No. 7, July 2021 (© 2021) pp. 1598–1618 10.1007/s10439-021-02784-1

The article 3D Printing for Cardiovascular Applications: From End-to-End Processes to Emerging Developments, written by Ramtin Gharleghi, Claire A. Dessalles, Ronil Lal, Sinead Mccraith, Kiran Sarathy, Nigel Jepson, James Otton, Abdul I. Barakat, and Susann Beier, was originally published electronically on the publisher’s internet portal on 17 May 2021 without open access. With the author(s)’ decision to opt for Open Choice the copyright of the article changed on 4 November 2021 to © The Author(s) 2021 and the article is forthwith distributed under a Creative Commons Attribution 4.0 International License, which permits use, sharing, adaptation, distribution and reproduction in any medium or format, as long as you give appropriate credit to the original author(s) and the source, provide a link to the Creative Commons licence, and indicate if changes were made.

The images or other third party material in this article are included in the article’s Creative Commons licence, unless indicated otherwise in a credit line to the material. If material is not included in the article’s Creative Commons licence and your intended use is not permitted by statutory regulation or exceeds the permitted use, you will need to obtain permission directly from the copyright holder. To view a copy of this licence, visit http://creativecommons.org/licenses/by/4.0/

The original article has been corrected.

